# The impact of public environmental concern on environmental pollution: The moderating effect of government environmental regulation

**DOI:** 10.1371/journal.pone.0290255

**Published:** 2023-08-17

**Authors:** Nengyu Liu, Yue Liu, Xiaofei Yu

**Affiliations:** 1 School of Economics, Zhejiang Gongshang University, Hangzhou, China; 2 Zhejiang Institute of Industry and Information Technology, Hangzhou, China; Shandong University, CHINA

## Abstract

As environmental problems continue to intensify, public environmental awareness and participation have become key forces in a modernized environmental governance system. Recognizing the importance of public participation in environmental governance, this study explores the influence of public pressure on environmental pollution and its implications for China’s long-term environmental management efforts. Using statistical and internet search data from 284 prefecture-level cities between 2011 and 2020, the study finds that a 1% increase in public environmental concern leads to a 0.009% reduction in pollution. The study also highlights the strengthening effect of government environmental regulation on the impact of public environmental concern. Moreover, regional heterogeneity analysis reveals a stronger effect of public environmental concern in cities facing low economic pressure. The findings of the study provide a reference for the construction of a coordinated and sustainable environmental governance model in China as well as in developing countries.

## 1. Introduction

China’s sustained economic growth has resulted in a range of issues, including excessive energy consumption, a rapid increase in pollution emissions, and a decline in environmental carrying capacity [1, 2]. While the Chinese government has implemented various monitoring measures to control environmental pollution [3, 4], the predominant governance approach has relied heavily on administrative means, involving direct intervention through the issuance of government administrative orders to address environmental pollution. Due to the rigid constraints on China’s administrative resources, relying solely on unilateral government administrative supervision can lead to inefficiencies and suboptimal outcomes in environmental regulation and governance. Drawing on the experiences of developed countries, a scientifically informed environmental governance system encompasses not only the government but also the participation of the general public [5]. Therefore, exploring the influence of public participation on environmental pollution holds significant implications for China’s long-term environmental management efforts.

The potential risks associated with environmental pollution have become an inherent driving force for public concern regarding environmental issues in China [6, 7]. The widespread use of mobile internet has enabled individuals to rapidly share and disseminate environmental-related information, enhancing public awareness and concern about environmental issues [8]. Additionally, a significant number of individuals express complaints about environmental pollution through social media and exert pressure on local governments to address these issues [9]. This has given rise to a bottom-up governance approach and an intangible constraint urging the government to address severe environmental problems [10]. While public attention plays a crucial role in improving urban environments in China, the full potential of this power has yet to be fully tapped.

In China, public participation mechanisms in environmental governance are still in the early stages of development. Public enthusiasm for environmental governance is influenced by various external factors, among which the regulatory and enforcement efforts of the government are crucial [7]. Despite the central government’s ongoing efforts to strengthen environmental protection, lax enforcement of environmental regulations remains a problem in some local governments. This issue is primarily attributed to the jurisdictional relationship between the central and local governments, as well as the promotion mechanisms for local officials, which prioritize economic development over environmental protection [11]. The lagging and insufficient government environmental regulation further weakens public environmental awareness and activism, subsequently impacting the overall ecological environment of society. Therefore, the regulatory role of government environmental supervision is a pertinent issue for discussion.

Using statistical and web search data from 284 prefectures in China between 2011 and 2020, this study discusses the impact of public environmental concern on environmental pollution and the moderating effect of government environmental regulation. The results show that public environmental concern can reduce environmental pollution levels, while government environmental regulation can enhance this effect. Furthermore, heterogeneity analysis shows that the weakening effect of public environmental concern on environmental pollution is more pronounced in cities with low economic pressure. This study highlights the need to incorporate public participation into China’s environmental governance system and to harness the power of the public to mitigate environmental pollution problems. In addition, this study highlights the importance of coordination between local government and public participation. These provide valuable insights for policy makers and practitioners in their efforts to achieve sustainable environmental management in China.

This study contributes to the literature in several ways. First, distinct from the conventional focus on the impact of government intervention on environmental pollution [12–15], this study places greater emphasis on the influence of public participation in addressing environmental pollution. We find that public pressure from the internet has made significant contributions to improving environmental pollution. Second, the role of local governments and public participation in the environmental governance system depends on their coordination [16]. However, existing research only focuses on the impact of one party on environmental pollution, neglecting the combined effects of both. This study addresses this research gap. Third, a closely related literature discusses the influence of public environmental concern on air pollution [17–19], yet there are potential endogeneity issues to be resolved, which may introduce substantial estimation biases. This study identifies an appropriate instrument variable (IV) to mitigate potential estimation biases.

The remainder of this study is organized as follows: Section 2 presents the research hypotheses and reviews the relevant literature. Section 3 introduces the data collection, empirical methods, and variables. Section 4 analyses and discusses the regression results. Section 5 provides the conclusions and policy implications and suggests future research directions.

## 2. Literature review and research hypotheses

Based on previous studies, this section first discusses the effects of public environmental concern on environmental pollution and discusses the intrinsic mechanisms at the individual, firm, and government levels. Second, this section summarizes the interplay between formal and informal regulation, i.e., the joint effect of government environmental regulation and public environmental concern. Finally, considering the economic differences among Chinese cities, we further sort out the heterogeneous manifestations of public environmental concern in cities of different economic levels.

### 2.1 Public environmental concern and environmental pollution

Environmental pollution is a multifaceted problem involving three key stakeholders: the public, corporate, and the government. First, public environmental concern reflects a tendency towards environmental protection [20]. The increase in environmental concern can enhance individuals’ motivation for pro-environmental behavior, such as reducing waste [21], minimizing energy consumption [22], and purchasing eco-friendly products [23, 24]. Moreover, environmental concern possesses the potential to yield a greater impact through the demonstration effect and collective action. As an increasing number of individuals become deeply invested in environmental issues, the overall level of environmental awareness and protection within society will undoubtedly escalate.

Second, the public environmental concern has the potential to influence corporate environmental behavior through both the commodity and capital markets. Specifically, the public can communicate their environmental values and preferences to businesses by supporting and purchasing environmentally-friendly products [25]. For instance, according to a survey conducted by Fauzan [26], public environmental concern is one of the most influential factors affecting the purchase of green products. Consequently, at the level of the commodity market, this incentivizes companies to proactively enhance their environmental management and emissions reduction measures in order to provide eco-friendly products that align with consumer expectations. In the capital market, the public’s environmental concern directly influences individual investment behavior. This impact is manifested as the public chooses to financially support environmentally-friendly companies while implementing "market punishment" towards environmentally unfriendly firms [27]. Such actions can exert direct economic pressure on companies in the capital market, compelling them to reconsider and improve their environmental management and emissions reduction measures.

Third, when public attention to environmental issues increases, governments are often under pressure to develop more stringent environmental protection policies. The government may consider implementing stricter emission standards, restricting pollutant emissions, promoting sustainable development, and other policy measures to meet the expectations and demands of the public for environmental protection [10, 28]. For example, Wang and Di [29] confirmed that the environmental governance preferences of local governments are influenced by complaints from residents within their jurisdiction. Based on the aforementioned arguments, this study proposes the following hypothesis:

**H1:** Public environmental concern can reduce environmental pollution.

### 2.2 The moderating effect of government environmental regulation

The environment possesses distinct characteristics of a public good, and when faced with environmental pollution issues, the public often attributes the responsibility of environmental governance to the government [30, 31]. Public environmental concern can be a social force for environmental improvement, and raising awareness of environmental issues can speed up the introduction of environmental measures [30]. The government, under the pressure of public opinion and responsibility, is likely to adopt stringent environmental regulatory actions, thus generating a positive demonstration effect [16]. Research indicates that public environmental awareness is not solely driven by individual factors but also influenced by the government’s inclination towards environmental protection [32]. Furthermore, government environmental supervision can bring environmental issues into the public spotlight through media attention and coverage, allowing more people to understand the severity and impact of environmental problems and motivating public concern and engagement in addressing them [7]. The theoretical study by Chen et al. [32] suggests that the interaction between the government and the public has a positive impact on environmental governance. Based on the aforementioned arguments, this study proposes the following hypothesis:

**H2:** Government environmental supervision can strengthen the influence of public environmental concern on environmental pollution.

### 2.3 Regional heterogeneity in the influence of public environmental concern

The Chinese government places significant emphasis on addressing climate change and advancing green, low-carbon development. Many cities have committed themselves to achieving the ambitious goals outlined in the Paris Agreement at the local level. Regrettably, not all cities are actively responding to this imperative. Under the combination of political centralization and economic decentralization in China, a promotion incentive system has emerged, wherein economic growth performance serves as the primary evaluation criterion [33]. Such a promotion race would incentivise local officials to improve economic performance, especially gross domestic product (GDP) and its growth rate [34], while ignoring environmental issue. Under this incentive system, local governments need to balance environmental protection with economic development. Although there is no absolute dichotomy between environmental protection and economic development, in practice, local governments’ motivation to combat environmental pollution is heavily influenced by the "Promotion Competitions" [35]. Some regions may be inclined to relax environmental standards and provide more opportunities for enterprises to develop, even ignoring environmental violations, in return for short-term economic growth and performance advantages. In cities with greater economic pressures, local governments tend to prioritize local economic development and selectively overlook social welfare indicators such as environmental quality, thus weakening their social responsibility for environmental protection [36]. This single incentive for promotion, while leading to rapid economic development, has also led to serious pollution, which has gradually developed into one of the most serious social problems [37]. Moreover, residents in such cities may be more concerned about economic and quality-of-life issues, with limited awareness and understanding of environmental pollution risks. For instance, a survey conducted by Yang et al. [38] indicates that in cities with better economic conditions, there is higher public engagement with environmental issues, leading to significant environmental improvement in those cities. These arguments give rise to two hypotheses:

**H3a:** In cities with lower economic pressures, the influence of public environmental concern on environmental pollution is stronger.
**H3b:** In cities with higher economic pressures, the influence of public environmental concern on environmental pollution is weaker.

**Fig 1:**
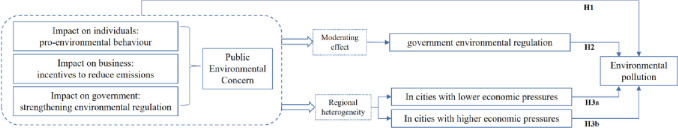
Analytical framework and research hypotheses.

Currently, environmental pollution governance includes both formal and informal regulation. A large body of literature has examined the impact of formal regulation, i.e. government regulation, on environmental pollution. In terms of informal regulation, previous studies have focused on the impact of public participation, social opinion and Non-Governmental Organizations on pollution, but the environmental concerns of the public have been neglected. Although, a number of studies have discussed the effects of a single form of regulation on environmental pollution, no study has yet explored the effects of the combined effects of formal planning and informal regulation. In addition, a number of studies have discussed the effect of economic differences on local preferences for pollution management, but it is unknown whether such differences affect the role of public environmental concerns. Based on the above theoretical analysis, we developed an analytical framework and research hypotheses, as shown in Fig 1.

## 3. Data source, methods, and variable description

### 3.1 Data source

The dataset used in this study was collected from multiple sources. First, panel data from 284 cities spanning the years 2011 to 2020 were collected from the statistical yearbooks of each city. Second, public environmental concern was measured using the Baidu Search Index, provided by China’s largest Chinese search engine. Third, government environmental penalty data were sourced from the Magic Weapon of Peking University, which collects administrative penalty information from across China. Finally, PM_2.5_ concentration data were compiled and calculated based on the annual average PM_2.5_ concentration data for global regions provided by the Socioeconomic Data and Applications Center at Columbia University.

### 3.2 Methods

To investigate the effect of public environmental concern on environmental pollution, this study constructs the following baseline regression model.

$$PI_{it}=\alpha +\beta PEC_{it}+\delta X_{it}+\mu_{i}+\upsilon_{t}+\varepsilon_{it}$$ (1)

where for city *i* and year *t*, *PI* refers to the level of pollution in the city. *PEC* denotes the public environmental concern. *X* represents control variables including economic development, industrial structure, population density, PM_2.5_ concentration, government investment in science and technology, and government investment in education. *μ*_*i*_ is an urban fixed effect to absorb all unique regional characteristics that do not change over time, such as cultural differences, and natural conditions. *υ*_*t*_ is a year fixed effect to capture the effects of unobservable variables that change over time, such as policy changes, and economic growth. *ε*_*it*_ is a random error term.

To further test the moderating effect of government environmental regulation, the following moderating effect model is constructed in this study. Model (2) represents the effects of public environmental concern, government environmental regulation and their interactions on environmental pollution.

$$PI_{it}=\alpha +\beta PEC_{it}+\lambda GER_{it}+\theta PEC_{it}*GER_{it}+\delta X_{it}+\mu_{i}+\upsilon_{t}+\varepsilon_{it}$$ (2)

where *GER* represents the strength of government environmental regulation. PEC*GER is the interaction effect of public environmental concern and government environmental regulation. The rest of the variables are consistent with those in Eq 1.

### 3.3 Variable description

#### 3.3.1 Dependent variable

*Environmental pollution (PI)*. In China, environmental pollution stems from various factors, with industrial emissions serving as the primary source of contamination. This includes industrial smoke, industrial sulfur dioxide, sulfur dioxide, and industrial wastewater [39]. Specifically, the release of industrial smoke into the atmosphere leads to the formation of suspended particulate matter, resulting in particle pollution. These particles have the capacity to adsorb harmful substances and disperse with airflow, negatively impacting atmospheric visibility and air quality [40]. Sulfur dioxide stands as a major contributor to air pollution. When the concentration of sulfur dioxide exceeds the stipulated limits in the atmosphere, it leads to a significant decline in environmental quality, posing severe health risks and potentially triggering natural disasters such as acid rain [41]. Industrial wastewater contains various chemical substances, heavy metals, and organic compounds. Discharging these pollutants into water bodies leads to water pollution, disrupting the equilibrium of aquatic ecosystems. Additionally, volatile organic compounds present in industrial wastewater can evaporate into the atmosphere. Once in the air, these compounds can participate in photochemical reactions, generating ozone and other harmful atmospheric pollutants, thereby contributing to the deterioration of air quality [42]. Existing literature commonly employs air pollution indicators as proxies for environmental pollution, but these indicators only reflect certain aspects of pollution and fail to provide a comprehensive representation of environmental pollution. Therefore, following the approach of Wan [43] and Zhang et al. [44], we fitted a comprehensive pollution index containing multiple pollution dimensions using three indicators: industrial smoke emissions, industrial sulfur dioxide emissions, and wastewater emissions, using the entropy weight method. The use of this index enhances the explanatory power and accuracy of the dependent variable.

The steps to calculate the comprehensive environmental pollution evaluation index using the entropy weighting method are as follows:

① Standardize the original data:

$$Y_{ij}=[X_{ij}-\min(X_{ij})]/[\max(X_{ij})-\min(X_{ij})]$$ (3)

where *Y*_*ij*_ is the standardised value of indicator *i* (*i* = 1, 2, …,n) for city *j* (*j* = 1, 2, …,m), *X*_*ij*_ is the original value of indicator *j* for city *i*.

② Calculate the entropy value of each indicator:

$$E_{j}=1/\ln(n)\sum_{i=1}^{n}P_{ij}\ln P_{ij}$$ (4)

where $P_{ij}=Y_{ij}/\sum_{i=1}^{n}Y_{ij}$, if *P*_*ij*_ = 0, then defined as *P*_*ij*_ln*P*_*ij*_ = 0.

③ Calculate each indicator weight:

$$W_{j}=(1-E_{j})/\sum_{j=1}^{m}\left(1-E_{j}\right)$$ (5)

④ Calculate *PI* according to the following formula:

$$PI_{i}=\sum_{j=1}^{m}W_{j}P_{ij}$$ (6)

#### 3.3.2 Independent variables

*Public environmental concern (PEC)*. With the development of the Internet, network search data that records the online behaviors of internet users can promptly capture the attention of market participants towards specific events, reflecting their preferences and behavioral intentions. According to study of Liu and Mu [45], we used the Baidu search index of the keyword "haze" to measure the degree of public concern about the environment. This was done for three reasons. First, since 2011, with the rapid development of social media in China, the issue of haze has been widely discussed by the public, and it has even sparked panic and concern among some individuals [46, 47]. Secondly, compared to other keywords related to environmental issues such as "environmental pollution" the term "haze" exhibits a relatively higher level of environmental awareness. This is because smog weather is characterized by its persistence, wide coverage, and perceptibility, which intuitively influences public perception of the environment. Additionally, the severity of smog can be readily perceived by the public through air visibility, leading to a more consistent perception among individuals. Therefore, public search information regarding smog accurately represents the overall level of public concern for the environment. Third, as the largest search engine in China, Baidu accounts for more than 90% of Chinese internet users, and using this index provides a comprehensive understanding of users’ search behavior. The data provide information on search frequency, time and location, which can be matched with other data available. In the robustness check, we used "environmental pollution" as a new keyword to remeasure public environmental concerns.

#### 3.3.3 Moderator variable

*Government Environmental Regulation (GER)*. We utilize the per capita number of environmental protection penalty cases at the city level as an indicator of local government regulatory efforts. In China, the environmental protection administrative departments impose administrative penalties on individuals or enterprises who violate environmental laws and regulations. The higher frequency of administrative penalties implemented by local governments indicates a greater emphasis on environmental protection and sustainable development in the respective regions, as well as a stronger degree of environmental governance [48].

#### 3.3.4 Control variables

To provide a more reliable estimation of the impact of public environmental concern on environmental pollution, this study incorporates a series of control variables following previous literature [10, 47]. These control variables include economic development (*LnGDP*), industrial structure (*Indstr*), population density (*LnPopden*), PM_2.5_ concentration (*LnMP*_*2*.*5*_), government investment in technology (*Invtec*), and government investment in education (*Invedu*). Table 1 presents the variable definitions and the summarized statistics.

**Table 1:** Variable description and summarized statistics.

Variable	Description	Min	Max	P25	P50	P75
*PI*	Synthetic indicators for the three-pollution data	-0.436	0.962	0.023	0.048	0.09
*PEC*	Public environmental concern	-1.86	6.085	2.004	3.264	4.003
*GER*	The logarithm of per capita administrative environmental penalty cases	-7.12	2.883	-3.317	-2.219	-1.178
*LnGDP*	The logarithm of Gross Domestic Product	4.896	10.228	6.723	7.231	7.901
*Indstr*	The ratio of value added in the tertiary sector to value added in the secondary sector	0.169	8.802	0.837	1.143	1.497
*LnPopden*	The logarithm of the ratio of population size to administrative area	2.944	7.362	5.446	5.916	6.351
*LnMP*_*2*.*5*_	The logarithm of the annual average PM2.5 concentration.	0.623	4.687	3.368	3.644	3.912
*Invtec*	The ratio of technology expenditure to total government expenditure	0.001	0.207	0.005	0.011	0.021
*Invedu*	The ratio of education expenditure to total government expenditure	0	0.356	0.151	0.176	0.203

## 4. Empirical results and discussion

### 4.1 Baseline results

Table 2 presents the estimation results of the baseline regression. Column (1) shows the results of the univariate analysis, indicating that the estimated coefficient of public environmental awareness is -0.009, which is statistically significant at the 1% level. In column (2), we include time and city fixed effects and the results do not change significantly. it strengthens the confidence in the observed relationship, as it indicates that the relationship holds even when accounting for the specific characteristics of each city and changes over time. In column (3), we include a range of important control variables, and the estimation results remain similar to those of the previous model. In column (4), we not only add rich control variables, but also consider time and city fixed effects. The results demonstrate that public environmental awareness contributes to a reduction in environmental pollution levels. This finding supports the research conducted by Long et al. [49].

**Table 2:** Regression results of the impact of public environmental concern on environmental pollution.

Variable	(1)	(2)	(3)	(4)
*PEC*	-0.009***	-0.01***	-0.006***	-0.009**
	(0.001)	(0.003)	(0.001)	(0.004)
*LnGDP*			0.030***	0.007
			(0.005)	(0.011)
*Indstr*			-0.009**	-0.001
			(0.003)	(0.004)
*LnPopden*			-0.018***	-0.009
			(0.006)	(0.022)
*LnMP*_*2*.*5*_			0.027***	0.034***
			(0.007)	(0.013)
*Invtec*			-0.094	0.029
			(0.126)	(0.123)
*Invedu*			0.068	0.169***
			(0.051)	(0.062)
Year FE	NO	YES	NO	YES
City FE	NO	YES	NO	YES
Constant	0.042***	0.045***	-0.166***	-0.111
	(0.003)	(0.003)	(0.034)	(0.141)
*R* ^2^	0.062	0.165	0.058	0.151
N	2,486	2,486	2,486	2,486

Note

* *p* < 0.1

** *p* < 0.05

*** *p* < 0.01; Standard errors in parentheses.

In recent years, the Chinese government has gradually transitioned from a traditional model of government-led environmental regulation and governance to a governance system characterized by shared responsibilities between the government and the public [16]. In this transition process, the Chinese government has started to pay attention to public environmental demands and continuously adjusts environmental governance policies based on information gathered from the internet and social media to understand public attitudes and opinions. Furthermore, widespread news coverage of environmental pollution issues on the Chinese internet reveals the severity and impact of environmental pollution, enhancing public awareness of the complexity of environmental problems and encouraging the Chinese public to take action to alleviate environmental pressures. The above conclusion is consistent with Hypothesis 1.

### 4.2 Robustness checks

To validate the reliability of our empirical analysis, we conducted a series of robustness tests.

First, endogeneity issues may exist between public environmental awareness and environmental pollution. For example, there could be a reciprocal causal relationship between public environmental awareness and pollution levels, where changes in pollution levels may affect public environmental awareness. To address the potential endogeneity problem, we employed educational level (*Edulev*) as an instrumental variable for public environmental awareness and conducted a two-stage least squares regression (2SLS) analysis. Previous studies have indicated that the level of public concern for environmental issues is influenced by their own educational attainment [45, 50]. Therefore, the instrumental variable is significantly related to public environmental awareness but has no direct impact on environmental pollution, satisfying the validity assumption of the instrumental variable. We utilized the number of university students enrolled in each city as a measure of educational level. The 2SLS results are presented in columns (1) and (2) of Table 3. The first-stage results show that the estimated coefficient for educational level is positive at a significance level of 1%, with an F value of 187.37, confirming the validity of the instrumental variable. The second-stage estimation results indicate that the coefficient of public environmental awareness is significantly negative at a 1% level, consistent with the previous estimates. This implies that our research findings hold true after overcoming endogeneity concerns.

**Table 3:** Results of robustness test estimation.

Variable	(1)	(2)	(3)	(4)
	First-stage	Two-stage	Replace the independent variable	Replace the dependent variable
*PEC*		-0.011***		-0.009***
		(0.003)		(0.003)
*PEC*_*2*_			-0.003*	
			(0.002)	
*Edulev*	0.289***			
	(0.048)			
*LNGDP*	0.988	0.182***	0.013*	0.012
	(0.087)	(0.031)	(0.008)	(0.011)
*Indstr*	-0.5466	-0.029***	-0.006	-0.003
	(0.07)	(0.005)	(0.004)	(0.005)
*LnPopden*	-0.210	0.162***	-0.010	-0.017
	(0.106)	(0.046)	(0.021)	(0.022)
*Invtec*	-8.255	-0.104	0.066	-0.053
	(3.665)	(0.185)	(0.106)	(0.140)
*Invedu*	-3.369	-0.228***	0.147***	0.159**
	(1.131)	(0.071)	(0.045)	(0.067)
*LnMP*_*2*.*5*_	0.155	0.094***	0.0213**	0.035**
	(0.148)	(0.021)	(0.01)	(0.014)
Constant			-0.1059	-0.103
			(0.131)	(0.144)
Year FE	YES	YES	YES	YES
City FE	YES	YES	YES	YES
F	183.37			
*R* ^*2*^			0.164	0.151
N	1,339	1,339	2,518	2,207

Note

* *p* < 0.1

** *p* < 0.05

*** *p* < 0.01; Standard errors in parentheses.

Second, considering the widespread use of mobile networks and smartphones in China, people have become more accustomed to using smartphones for daily work and information retrieval. Therefore, we replaced the independent variable with a new measure of public environmental awareness, namely, the search volume on the mobile platform (*PEC*_*2*_), and re-estimated the regression model as presented in column (2) of Table 3.

Third, considering that the governance of environmental pollution requires a certain time lag, the impact of public environmental awareness on pollution may exhibit lagged effects. Therefore, we included the lagged environmental pollution variable as the new dependent variable and re-estimated model (1). The results are presented in column (3) of Table 3. The above results are consistent with the baseline results, indicating the robustness and reliability of our findings.

### 4.3 The moderating role of government environmental regulation

**Fig 2:**
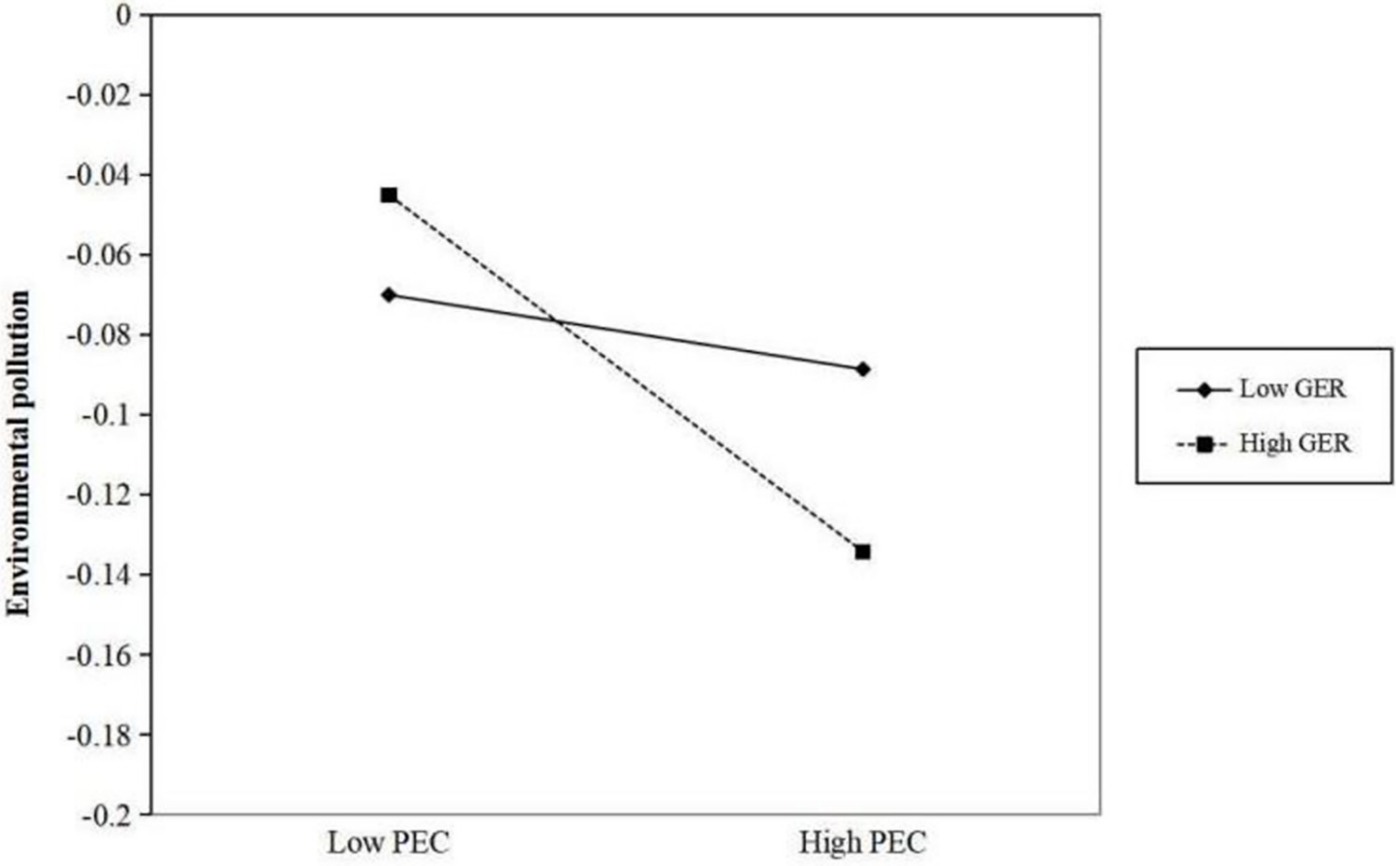
The interaction between public environmental concern and government environmental regulation on environmental pollution.

To examine the moderating role of government environmental regulation, we employ Eq 2. To provide a more intuitive demonstration of the moderating effect of government environmental regulation, we adopt the approach used by Dawson [51]. Specifically, we divide the government environmental regulation variable into high and low groups based on one standard deviation above and below the mean. We then conduct a simple slope test and create an effect analysis graph. As shown in Fig 2, when the intensity of government environmental regulation increases, the mitigating effect of public environmental concern on environmental pollution becomes stronger (interaction term: *θ* = -0.001, t = -2.61, p = 0.01). This finding complements the literature on the impact of government environmental regulation. The findings of Zhao et al [7] share some similarities with ours, suggesting that the low efficiency of environmental regulatory enforcement in China hampers public engagement in environmental governance and fails to exert a positive moderating effect on corporate green behaviour. In summary, Hypothesis 2 is supported.

### 4.4 Regional heterogeneity

Considering that the level of economic development may influence local environmental governance and public concern preferences, we analyse the heterogeneity across cities with different economic levels in this section. We use the median of cities’ GDP as the criterion for classification, with cities above the median categorized as low economic pressure and those below the median as high economic pressure. The estimation results are presented in Table 4. As expected, the impact of public environmental concern on environmental pollution is more significant in cities with low economic pressure. Therefore, we find support for research hypotheses H3a and H3b.

**Table 4:** Results of regional heterogeneity.

Variable	(1)	(2)
	High economic pressure	Low economic pressure
*PEC*	-0.007*	-0.011**
	(0.004)	(0.005)
*LNGDP*	0.002	-0.001
	(0.011)	(0.025)
*Indstr*	-0.004	0.009
	(0.006)	(0.008)
*LnPopden*	0.013	-0.058
	(0.027)	(0.036)
*Invtec*	0.034**	0.037
	(0.014)	(0.023)
*Invedu*	-0.055	0.054
	(0.107)	(0.193)
*LnMP*_*2*.*5*_	0.110**	0.114
	(0.046)	(0.115)
Year FE	YES	YES
City FE	YES	YES
Constant	-0.195	0.253
	(0.144)	(0.321)
*R* ^2^	0.113	0.249
*N*	1,212	1,274

Note

* *p* < 0.1

** *p* < 0.05

*** *p* < 0.01; Standard errors in parentheses.

## 5. Conclusion

Strengthening ecological environmental protection requires effective public participation. China is actively establishing and improving systems for public participation in environmental management and decision-making, which not only enhances public awareness of environmental protection but also urges the government to adopt effective environmental management strategies. Especially with the rapid development of modern media, an increasing number of individuals are consciously and systematically paying attention to pollution issues and strongly demanding environmental governance. This widespread dissemination and communication enable the public to better focus on environmental issues, generate collective action, and drive the progress of environmental governance. This bottom-up mechanism has become an indispensable and important component of China’s current environmental pollution control system, promoting the reduction of pollution levels.

The study highlights the role of public environmental concerns arising from the widespread use of mobile internet and social media in driving public demand for improved environmental conditions. This study utilizes statistical data and internet search data from 284 prefecture-level cities in China between 2011 and 2020 to empirically examine the impact of public environmental concern on environmental pollution, with government environmental regulation as a moderating factor. The study draws three main conclusions. First, public environmental concern can effectively reduce environmental pollution. Specifically, a 1% increase in public environmental concern leads to a 0.009% reduction in environmental pollution. Even after addressing endogeneity concerns, the causal relationship between public environmental concern and environmental pollution persists. Second, government environmental regulation strengthens the impact of public environmental concern on environmental pollution. Finally, the analysis of regional heterogeneity suggests that in cities facing low economic pressure, public environmental concern has a stronger effect on reducing environmental pollution.

This research has broad significance and policy implications. First, researchers and policymakers should pay attention to public demands regarding environmental issues and guide public environmental concern in a rational manner. They should fully leverage the noncoercive constraints imposed by the public on businesses and local governments in environmental development. Second, governments should strengthen the capacity and efficacy of environmental regulatory institutions to ensure the effective implementation and enforcement of environmental regulations. Increasing penalties for environmental violations and enhancing the transparency and fairness of environmental regulation can enhance public trust and support for government environmental actions. Third, governments need to promote the coordinated development of the economy and the environment, especially in economically disadvantaged areas. This can be achieved through incentive measures and tax policies to encourage businesses to adopt environmentally friendly technologies and measures, reduce environmental pollution emissions, and actively participate in environmental protection efforts.

Despite the informative results, this study has certain limitations. First, it only considers the macrolevel impact of public environmental concern on environmental pollution. However, the impact of changes in environmental concern at the individual level on environmental behaviour is not examined. Further research can explore this aspect with available data. Additionally, although we discuss the moderating role of government environmental regulation, we do not consider the effects of government environmental incentives. Future research can delve into the mechanisms of different types of government behaviour as regulatory factors.

## Supporting information

S1 Data (XLSX)
